# *ADRB3* Polymorphism Associated with BMI Gain in Japanese Men

**DOI:** 10.1155/2012/973561

**Published:** 2012-04-08

**Authors:** Shouhei Takeuchi, Takahiko Katoh, Takenori Yamauchi, Yoshiki Kuroda

**Affiliations:** ^1^Department of Public Health, Faculty of Medicine, University of Miyazaki, 5200 Kihara, Kiyotake, Miyazaki 889-1692, Japan; ^2^Department of Public Health, Faculty of Life Sciences, Kumamoto University, 1-1-1 Honjou, Kumamoto 860-8556, Japan

## Abstract

*Objective*. The aim of this study was to evaluate the association between the Trp64Arg polymorphism in the beta3-adrenergic receptor gene (*ADRB3*: rs4994) and BMI and serological and anthropometric data in healthy Japanese. *Methods*. Healthy Japanese recruited in a large-scale integrated manufacturing facility in Japan (*N* = 1355; age: 37.25 ± 9.43; BMI: 22.86 ± 3.46) were eligible for analysis. The anthropometric data and serological data were measured during a comprehensive health check, and a self-reporting questionnaire was used to assess lifestyle habits (current exercise, smoking status, alcohol intake, and working style) and weight at age 20. Genotyping for the *ADRB3* polymorphism was performed by PCR-RFLP method. *Results*. Among 1355 participants, the genotype frequencies of the Trp/Trp, Trp/Arg, and Arg/Arg variants were 920 (67.9%), 394 (29.1%), and 41 (3.05%), respectively. In the multivariate analysis, a multiple linear regression model in men for the adjustment of age, drinking habits, smoking habits, exercise habits, working status and serological measurements statistically showed an overall weak significance between annual BMI gain from age 20 and age, LDL or *ADRB3* polymorphism. *Conclusions*. The level of LDL, age, and *ADRB3* polymorphism (Arg/Arg genotype) were statistically associated with annual BMI gain in Japanese men.

## 1. Introduction

The beta3-adrenergic receptor (*ADRB3*) is primarily expressed in adipose tissue [1] and is involved in the regulation of energy metabolism. In 1995, three reports related to the polymorphism of *ADRB3* in codon 64 (Trp64Arg: rs4994) were published. They reported that early development of type 2 diabetes mellitus, lower resting metabolic rate, abdominal obesity, and resistance to insulin were associated with persons homozygous for the variant allele (Arg/Arg) compared to those homozygous for the wild-type allele (Trp/Trp) and those heterozygous for the variant allele (Trp/Arg) [2–6]. Piétri-Rouxel et al. showed that in human HEK293 cells with *ADRB3* variant allele, the reduction in cAMP accumulation in response to beta3-adrenergic agonists resulted in decreased lipolysis and thermogenesis [7]. Umekawa et al. also showed that *ADRB3* variant was associated with lower lipolytic activities in human omental adipocytes [8]. Therefore, an impairment of *ADRB3* may lead to obesity through the energy expenditure reduction of fat tissue. This allowed us to accept *ADRB3* as a candidate gene for obesity [9, 10].

 However, the association between BMI gain and the *ADRB3* polymorphism is still controversial. The meta-analyses indicated that there was an association between BMI and the *ADRB3* polymorphism among an obese population [11] and among obese and normal populations [12]. Conversely, several studies [13, 14] suggested that there was no association between BMI and the *ADRB3* polymorphism. The aim of this study was to evaluate the influence of *ADRB3* polymorphism on BMI and serological and anthropometric data in healthy Japanese.

## 2. Subjects and Methods

### 2.1. Subjects

 The participants were recruited from employees working at a large-scale integrated manufacturing facility in Japan, and their routine medical checkup data were used for the analysis. Of the 1401 participants, only the workers who were out of work or who had serious symptoms were excluded from this study, 1355 agreed to participate in the present study with informed consent, 1074 (77%) were males and 284 (20%) were females. We analyzed the *ADRB3* polymorphism, lifestyle habits, and anthropometric data (2 men and a woman had missing data) for the participants. Their ages ranged from 18 to 65 years (mean: 37.3 ± 9.4 years). During a comprehensive health check, the following anthropometric data and serological data were measured: height, weight, systolic blood pressure (SBP), diastolic blood pressure (DBP), GOT, GPT, *γ*-GTP, total cholesterol, low-density lipoprotein cholesterol (LDL) and high-density lipoprotein cholesterol (HDL), and triglyceride. For the lifestyle assessment, current exercise (no, 1-2 days/week, or 3–7 days/week), smoking status (no, 1–20 cigarettes/day, or more than 20 cigarettes/day), alcohol intake (no, sometimes, or every day), working style (having night shift or not), and weight at age 20 were obtained through a self-reporting questionnaire. Body mass index (BMI) was calculated as weight (in kilograms) divided by height (in meters) squared. We followed the Ethical Guideline for Human Genome/Gene Analysis Research endorsed by the Japanese government. The study protocol was approved by the ethics committee of University of Miyazaki Graduate School of Medical Sciences, and all subjects provided written informed consent.

### 2.2. Genotyping

 Genomic DNA was extracted from peripheral blood leukocytes by using a DNA Extractor WB Kit (Wako Pure Chemical Industries, Osaka, Japan). Amplification of DNA by polymerase chain reaction was carried out to analyze for the Trp64Arg polymorphism with the primer (sense: 5′-CGCCCAATACCGCCAACAC-3′, antisense: 5′-CCACCAGGAGTCCCATCACC-3′) under the same procedure described by Widen et al. with the restriction enzyme Mva I [4]. The amplified fragments of 210 bp were digested with Mva I and analyzed by 3% agarose-gel electrophoresis. The fragment size was judged as the Arg/Arg type if 161 base pairs; the Trp/Trp type if 99 and 62 base pairs; the Trp/Arg type if 161, 99, and 62 base pairs.

### 2.3. Statistical Analysis

 Results are presented as mean ± standard deviation (SD), and categorical variables are expressed as numbers counted. All analyses were performed stratified by sex due to the difference in number of male and female participants. The exact test was used to verify Hardy-Weinberg equilibrium of genotype frequencies. The analysis of variance (ANOVA) or Kruskal-Wallis rank sum test was used to access the differences among *ADRB3* gene polymorphism. Multivariate analysis for adjusting age, sex, current exercise, alcohol intake, working style, and smoking habits was performed by multiple linear regression analysis. The level of statistical significance was set at *P* < .05. All analyses were performed using R version 2.12.2.

## 3. Results

 Genotyping of the *ADRB3* polymorphism in the 1355 participants showed that those homozygous for the wild-type allele (Trp/Trp) were 920 (67.9%), those heterozygous for the variant allele (Arg/Trp) were 394 (29.1%), and those homozygous for the variant allele (Arg/Arg) were 41 (3.0%). These results were in the Hardy-Weinberg equilibrium and did not conflict with the results previously reported in another Japanese population (*P* = .93).

 The demographic characteristics and lifestyle habits of the participants stratified by the genotype of the *ADRB3* polymorphism are presented in Table 1. Among all participants, the mean age was 37.25 ± 9.43 (mean ± SD) and the mean BMI was 22.86 ± 3.46 (mean ± SD). There were no significant differences with age and BMI in each stratified group. We also divided participant data into categories based on sex, smoking status, current exercise, and working style. In each category, the distributions of the genotypes were not different from the results previously reported in another Japanese population.

The serological test results of the participants are shown in Table 2. There was no association between these serological results and the genotype of the *ADRB3* polymorphism (ANOVA and Kruskal-Wallis rank sum test).

A multiple linear regression model for the adjustment of age, sex, alcohol intake, smoking status, current exercise, working status, LDL, and *ADRB3* polymorphism (Table 3) showed an overall weak model for predicting annual BMI gain (the degree of BMI increase) from age 20 per year by age, smoking status, LDL, and Arg/Arg genotype. To avoid the bias caused by the difference in number of male and female participants, data were stratified by sex and analyzed separately. There were statistically significant associations in male between the genotype of the *ADRB3* polymorphism, age, and LDL and the annual BMI gain (Table 4), and there was no statistically significant association in female (Table 5).

## 4. Discussion

 The effects of *ADRB3* polymorphism were reported in 1995 [2–4]. The potential for weight gain among the population having the Trp/Arg mutation of the *ADRB3* gene has been suggested [2]. It was thought that the variant allele (Arg/Arg) had lower lipolytic activities and induced heavier weight [15]. In the present study, we analyzed the relationship between annual BMI gain and the *ADRB3* polymorphism in Japanese young men who are generally healthy. In the multivariate analysis, the *ADRB3* genotype (Arg/Arg), age, and LDL were associated with annual BMI gain. This result indicated that the annual BMI gain was increased with Arg/Arg genotype compared to Trp/Trp genotype, and high LDL was also related to increased annual BMI gain. The high LDL, however, could not be thought to be the cause of the annual BMI gain. Therefore, the high LDL was thought to be a result of increased body weight. 

 On the other hand, our results indicated that the *ADRB3 *polymorphism was not associated with current BMI. The relationship between the *ADRB3* polymorphism and current BMI has been controversial. Shiwaku et al. [16], Tahara et al. [17], Witchel et al. [18], and Matsushita et al. [14] reported that the *ADRB3 *polymorphism and BMI have not associated significantly with each other. Conversely, Oizumi et al. [15] pointed out that the Arg/Arg genotype was associated with obesity and current BMI. There were meta-analysis studies concerning the *ADRB3* polymorphism and obesity [11, 12] which indicated that Arg/Arg genotype led to increased body weight. These previous studies seem to lead the hypothesis that the *ADRB3* polymorphism is associated with current BMI and suggest that the *ADRB3* polymorphism could be a genetic predictor of increased body weight. However, there still have been confounding factors that induced obesity and overweight such as lack of exercise, over intake of energy, and predisposition of other genes. These confounders could be one reason why the relationship between the *ADRB3* polymorphism and BMI is still controversial. 

 While Matsuo et al. [19] indicated that BMI for older adults (60–79-year-age group) was higher than that of middle-aged adults (40–59-year-age group), other reports showed that BMI gain is more severe in middle age than old age [20–22]. Berg et al. [20] indicated that the largest increases in obesity and overweight were in young people. On the other hand, the effects of genetic predisposition on difference in body weight might exist to a greater extent among middle aged than older adults [23]. The present study also indicated that annual BMI gain was greater with Arg/Arg genotype compared to Trp/Trp in younger men (the mean age was 37.25 ± 9.43). Matsushita et al. [14] suggested little or no influence of the *ADRB3* polymorphism on BMI gain in their report. However their mean age was older than that of the present study. BMI gain (the degree of BMI increase) might be thought to be more suitable to evaluate the relation between the *ADRB3* polymorphism and body weight influence, and the time for evaluation of BMI gain related to the *ADRB3* polymorphism was more suitable in the younger group. 

 In the present study, we had some limitations. One of them was that our data might include the information bias because we used the questionnaire to get the weight at age 20. We believed that the body weights in the questionnaire were not so different from true weight at age 20 because many people can remember their weight at age 20. However, caution was needed in evaluating this data for female participants, as women tended to reply lighter weight. Another was the lack of checking the plasma glucose, HbA1c, and fasting insulin data, even NIDDM was discussed in this article. 

 Obesity and overweight are associated with incidence of diabetes. The Trp/Arg genotype of the *ADRB3* polymorphism has been said to be associated with resistance to insulin and may contribute to the early onset of NIDDM [3, 4]. We have not evaluated the relationship between the incidence of diabetes and the *ADRB3* polymorphism. Considering that Sasai et al. [24] showed that the effects of BMI on diabetes mellitus are greater among middle-aged than older adults, and the present result also suggests that the Arg/Arg genotype of *ADRB3* polymorphism might be a risk factor for diabetes. 

 We also have not evaluated the effects of exercise in relation to the *ADRB3* polymorphism. The relationship between the *ADRB3* polymorphism and BMI change (gain or reduction) with health programs, including training or exercise, has been controversial as well. The resistance to BMI reduction in those who had the variant allele of *ADRB3* after exercise or training was suggested by the ratio of visceral to subcutaneous fat area after a 3-month weight reduction intervention among women in their 40s and 50s [25]. However, no resistance to BMI reduction in peoples who had the variant allele of *ADRB3* was reported [17, 26–28]. As an important theme regarding the *ADRB3* polymorphism, further investigation into the relationship between *ADRB3* and the effects of exercise on obesity is warranted.

## 5. Conclusion

 In conclusion, *ADRB3* polymorphism and high levels of LDL were associated with annual BMI gain in men. On the other hand, the present study did not indicate the same relationship with *ADRB3* polymorphism in women. Further studies are needed to clarify the underlying mechanism between the *ADRB3* polymorphism and annual BMI gain and to evaluate the relationship between the effect of exercise and the *ADRB3* polymorphism.

## Figures and Tables

**Table 1:** Sociodemographic information and lifestyle habits stratified by *ADRB3* polymorphism.

		Trp64Trp	Trp64Arg	Arg64Arg
*N*		920	394	41
Age		37.31 ± 9.29	37.27 ± 9.66	35.61 ± 10.38
Sex	Male	719	320	33
	Female	201	74	8
BMI (kg/m^2^)		22.86 ± 3.49	22.91 ± 3.44	22.28 ± 2.88
Smoking status	No	450	191	15
	≦20 cigarettes	381	172	20
	≧21 cigarettes	89	31	6
Current exercise	No	591	217	25
	1-2 days/week	261	144	12
	3–7 days/week	68	33	4
Working style	Having shift work	489	211	25
	Not having shift work	431	183	16

All variables did not associate with the *ADRB3* polymorphism.

**Table 2:** Serological variables stratified by the *ADRB3* polymorphism.

	Trp64Trp	Trp64Arg	Arg64Arg
SBP (mmHg)	121.93 ± 14.47	122.23 ± 13.82	118.24 ± 12.17
DBP (mmHg)	73.72 ± 11.17	74.27 ± 11.74	72.78 ± 8.6
GOT (IU/l)	21.51 ± 10.52	21.37 ± 9.13	20.73 ± 8.54
GPT (IU/l)	29.32 ± 23.91	29.47 ± 20.5	26.49 ± 16.42
LDL (mg/dL)	111.57 ± 30.64	111.85 ± 32.5	105.66 ± 27.55
HDL (mg/dL)	58.64 ± 13.79	59.54 ± 14.79	56.16 ± 11.9
TG (mg/dL)	139.6 ± 126.84	138.48 ± 116.33	138.1 ± 84.91

No significant difference among the *ADRB3* polymorphism was found in each serological variable.

**Table 3:** Multiple regression analysis for annual BMI gain and variables.

	Estimate	SE	*t* value	Pr(>|*t*|)
Intercept	0.171	0.096	1.788	0.075
Age	−0.000	0.000	−2.241	0.026*
Sex	0.014	0.060	0.233	0.816
Alcohol intake	0.000	0.000	−0.286	0.776
Current exercise	−0.000	0.028	−0.338	0.736
Working style	0.013	0.037	0.359	0.720
Smoking status	−0.057	0.028	−2.053	0.041*
*ADRB3* (Trp/Arg)	−0.000	0.037	−0.078	0.938
*ADRB3* (Arg/Arg)	0.216	0.082	2.626	0.009**
LDL	0.000	0.000	3.449	<0.001**

From the stepwise multiple regression analysis, age, sex, alcohol intake, current exercise, working style, smoking status, LDL, and the *ADRB3* polymorphism were selected for the final model of the predictor of BMI gain, adjusted *R* ^2^ = .0796 (*F*-statistic = 2.874, *P* < .01).

**Table 4:** Multiple regression analysis for annual BMI gain and variables in Japanese men.

	Estimate	SE	*t* value	Pr(>|*t*|)
Intercept	0.193	0.097	1.998	0.047*
Age	−0.005	0.002	−2.326	0.021*
Alcohol intake	0.000	0.000	−0.649	0.517
Current exercise	0.002	0.027	0.067	0.947
Working style	0.001	0.038	0.015	0.988
Smoking status	−0.053	0.028	−1.883	0.061
*ADRB3* (Trp/Arg)	0.009	0.038	0.227	0.821
*ADRB3* (Arg/Arg)	0.218	0.079	2.761	0.006**
LDL	0.002	0.001	3.245	0.001**

From the stepwise multiple regression analysis, age, alcohol intake, current exercise, working style, smoking status, LDL, and the *ADRB3* polymorphism were selected for the final model of the predictor of BMI gain, adjusted *R* ^2^ = .092 (*F*-statistic = 3.224, *P* < .01).

**Table 5:** Multiple regression analysis for annual BMI gain and variables in Japanese women.

	Estimate	SE	*t* value	Pr(>|*t*|)
Intercept	−0.421	0.474	−0.888	0.392
Age	0.004	0.012	0.363	0.723
Alcohol intake	0.000	0.000	0.486	0.636
Current exercise	−0.357	0.263	−1.356	0.200
Working style	0.147	0.153	0.958	0.357
Smoking status	0.057	0.183	0.309	0.763
*ADRB3* (Trp/Arg)	0.127	0.256	0.497	0.628
LDL	0.005	0.003	1.746	0.106

From the stepwise multiple regression analysis, age, alcohol intake, current exercise, working style, smoking status, LDL, and the *ADRB3* polymorphism were selected for the final model of the predictor of BMI gain, adjusted *R* ^2^ = −.078 (*F*-statistic = .803, *P* > .05).
